# Mechanism exploration of Osteoking in the treatment of lumbar disc herniation based on network pharmacology and molecular docking

**DOI:** 10.1186/s13018-024-04570-w

**Published:** 2024-01-24

**Authors:** Xinlei Luo, Jingjing Liu, Xiaoxi Wang, Qiaojun Chen, Yanfa Lei, Zewei He, Xiaowei Wang, Yan Ye, Qiang Na, Changtao Lao, Zhengchang Yang, Jun Jiang

**Affiliations:** 1 Department of Spinal surgery, Southern Central Hospital of Yunnan Province, Honghe, China; 2 Department of Orthopedics, The Sixth Affiliated Hospital of Kunming Medical University, Yuxi, China

**Keywords:** Osteoking, Lumbar disc herniation, Network pharmacology, Molecular docking

## Abstract

**Objective:**

Lumbar disc herniation (LDH) is a common spinal surgical disease. Low back and leg pain caused by LDH is the main factor leading to functional disability, which has caused a serious burden to patients and society. Osteoking can delay the progression of osteoporosis and osteoarthritis, and even has a significant effect on the prevention of deep vein thrombosis after fracture surgery. In recent years, it has been gradually used in the treatment of LDH and has received significant results. However, the underlying mechanism remains unclear. The aim of this study was to predict the mechanism of Osteoking in the treatment of LDH through network pharmacology and verify it by molecular docking method.

**Methods:**

The TCMSP database was used to collect the relevant active components and targets of Osteoking, while the GeneCards, OMIM and DisGeNET databases were utilized to collect the relevant disease targets of LDH. The Venny 2.1.0 software was employed to obtain the intersecting gene targets of Osteoking and LDH. PPI network construction and core target selection were performed using Cytoscape 3.9.0 software. The Metascape database was used for GO and KEGG enrichment analysis of the relevant targets. Finally, molecular docking was conducted using AutoDock software.

**Results:**

The study identified 116 potential targets and 26 core targets for the treatment of LDH with Osteoking. Pathways in cancer, Alzheimer's disease, microRNAs in cancer and the IL-17 signalling pathway were among the main involved signalling pathways. Molecular docking results demonstrated that the key targets AKT1, IL-6, ALB, TNF and IL-1β exhibited relatively stable binding activities with the main active components of Osteoking.

**Conclusions:**

Osteoking can alleviate the symptoms of lumbar disc herniation through the modulation of multiple targets and signalling pathways.

## Introduction

Lumbar disc herniation (LDH) is a common disease in spinal surgery, which often occurs in young adults [1, 2], and its symptoms of low back and leg pain are the main factors leading to functional disability [3, 4]. Moreover, the survey shows that in the US, it costs more than 100 billion dollars to treat the pain caused by lumbar disc herniation every year [5], which has caused a serious burden to the sick people and society. Intervertebral disc is composed of nucleus pulposus, annulus fibrosus and cartilage endplate, which is the largest avascular nerve tissue in human body. The nutrient supply of intervertebral disc cells is mainly obtained by passive diffusion at the cartilage endplate with rich blood vessels. This microenvironment makes the intervertebral disc cells lack nutrition and vitality, and is in an anoxic state [6]. When the intervertebral disc degenerates, the degradation of extracellular matrix leads to the degeneration of nucleus pulposus [7], and type II collagen is gradually replaced by type I collagen, the water loss and elasticity of the intervertebral disc decrease. Finally, the nucleus pulposus breaks through the fibrous ring and protrudes outwards due to uneven stress, causing a series of complications [8]. More and more attention has been paid to the relationship between LDH and immune response. Many immune mediators in LDH are highly expressed. Intervertebral disc is an immune privileged organ, and NP is isolated from immune system due to its special structure. When NP protrudes, the immune response is triggered, which plays a crucial role in disc degeneration, and the infiltration of immune cells accelerates the degeneration of intervertebral disc [9]. Interleukin-23/Th17 axis is a newly discovered immune axis in recent years. When the nucleus pulposus is exposed to the immune system, its self-antigen may promote the differentiation of T cells on its surface into T17 cells, thereby producing cytokines such as interleukin-17, interleukin-23 and tumour necrosis factor-a, which mediate inflammatory reactions and autoimmune reactions, promote intervertebral disc degeneration and cause pain symptoms. In addition, the imbalance of human immune microenvironment can promote inflammatory response and nucleus pulposus cell death, leading to intervertebral disc degeneration and herniation.

Osteoking, also known as Henggu Bone Healing Agent, is produced in Yunnan Province, China, and is mainly made of traditional Chinese medicines such as Panax notoginseng, Radix Astragali and Ginseng, with a long history [10]. Studies have shown that Osteoking can delay the progress of osteoporosis and osteoarthritis through TGF-β and other signal pathways, and even reduce the incidence of deep vein thrombosis after intertrochanteric fracture of femur, with remarkable curative effect [11–14]. With the deepening of research on Osteoking, its role has also been explored. Nowadays, it is gradually used to treat LDH in clinic and has received significant curative effect. Unfortunately, its mechanism of action has not yet been elucidated. Therefore, the aim of the present study was to predict the mechanism of Osteoking in the treatment of LDH by network pharmacology and verify its reliability by molecular docking, so as to provide reference for clinical practice and scientific research.

## Methods

### Screening of active components and targets of Osteoking

The main components of Osteoking (Tangerine Peel, Safflower, Radix Astragali, Notoginseng, Ginseng, Eucommia Bark and Datura Flower) were searched using TCMSP database (https://old.tcmsp-e.com/tcmsp.php). The active components of the drug were screened out with an oral bioavailability (OB) of ≥ 30% and a drug-like property (DL) of ≥ 0.18. The SMILES numbers of the active components were obtained from PubChem database (https://pubchem.ncbi.nlm.nih.gov), and the targets corresponding to the active components were retrieved from SwissTargetPrediction database (http://www.swisstargeting.ch).

### Screening of targets for LDH

The databases of GeneCards (https://www.genecards.org), OMIM (https://www.omim.org) and Disgene (https://www.disgenet.org) were used to search for the related targets of LDH, with the search term "lumbar disc herniation". Collate the obtained targets and delete the duplicates.

### Screening of potential targets

The intersection targets of drug targets and disease targets were obtained by Venny 2.1.0 software, and the Venn diagram was produced. The intersection targets were the potential targets.

### Construction of protein–protein interaction (PPI) network

A String database (https://cn.string-db.org) was used to construct the protein–protein interaction network model of potential targets. Set the species as "Homo sapiens" and the minimum interaction score as 0.9 for screening. The obtained data were imported into Cytoscape 3.9.0 software. The built-in software (Centiscape 2.2) was used to calculate the network topology parameters, and the core targets were screened out according to the closeness, between ness and degree.

### Construction of drug-active ingredient-target network diagram

The composition data were imported into Cytoscape 3.9.0 software to produce a drug-active ingredient-target network diagram, and topology analysis was performed to screen the main active ingredients of the drug according to the degree value.

### KEGG and GO enrichment analysis

The Metascape database (https://metascape.org) was used for KEGG and GO enrichment analysis of potential targets. Among them, GO analysis included biological process (BP), cell components (CC) and molecular function (MF) analysis, with *P* < 0.01. The data of the first 20 analysed items were imported into bioinformatics database (http://bioinformatics.com.cn) to produce a bubble chart.

### Molecular docking

The 2D structural diagrams of the first three components of the main active components (sorted according to degree value) were obtained using TCMSP database and PubChem database, and imported into Chem3D software to minimize energy and prepare small-molecule ligands. The protein structures of the first five targets (sequenced according to degree value) of the core targets were obtained using PDB database (https://www.rcsb.org), and protein receptors were prepared by removing water molecules and ligands using PyMOL software. Finally, the molecular docking of the ligand and the receptor was realized by using AutoDock software.

### Statistical analysis

Metascape database was used for GO and KEGG pathway enrichment analysis. The GO and KEGG enriched terms were collected for biological process (BP), cell component (CC) and molecular function (MF), at a cutoff of *p* < 0.01.

## Results

### Screening of potential targets for drugs and diseases

**Fig. 1:**
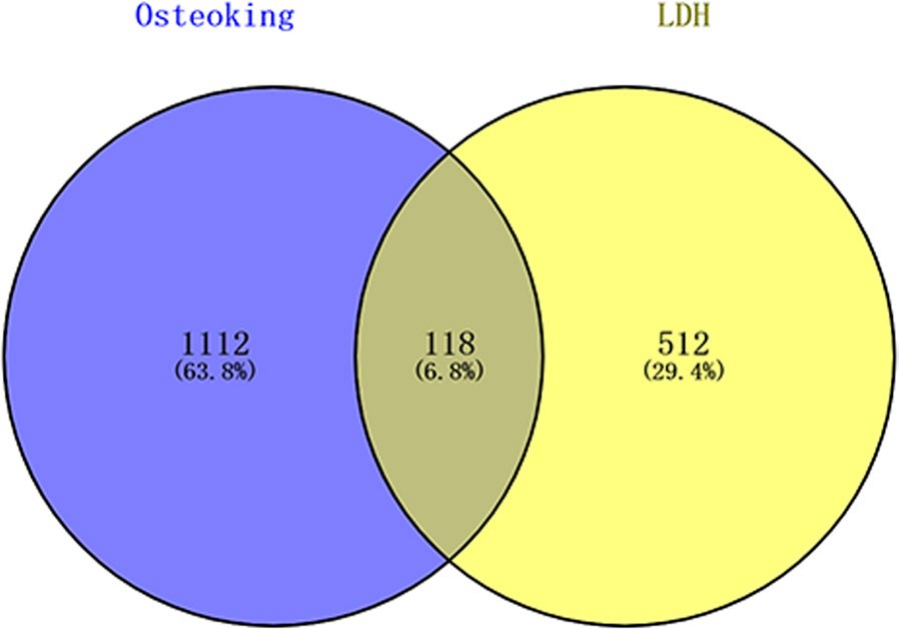
Venn diagram showing the overlapping target genes for Osteoking against LDH. LDH, Lumbar disc herniation

A total of 1230 targets and 114 active components of Osteoking were retrieved through the TCMSP database. A total of 630 disease targets for LDH were retrieved from the GeneCards, OMIM and DisGeNET databases. A total of 118 potential targets were obtained by intersection with Venny diagram (Fig. 1).

### Construction of protein–protein interaction (PPI) network

**Fig. 2:**
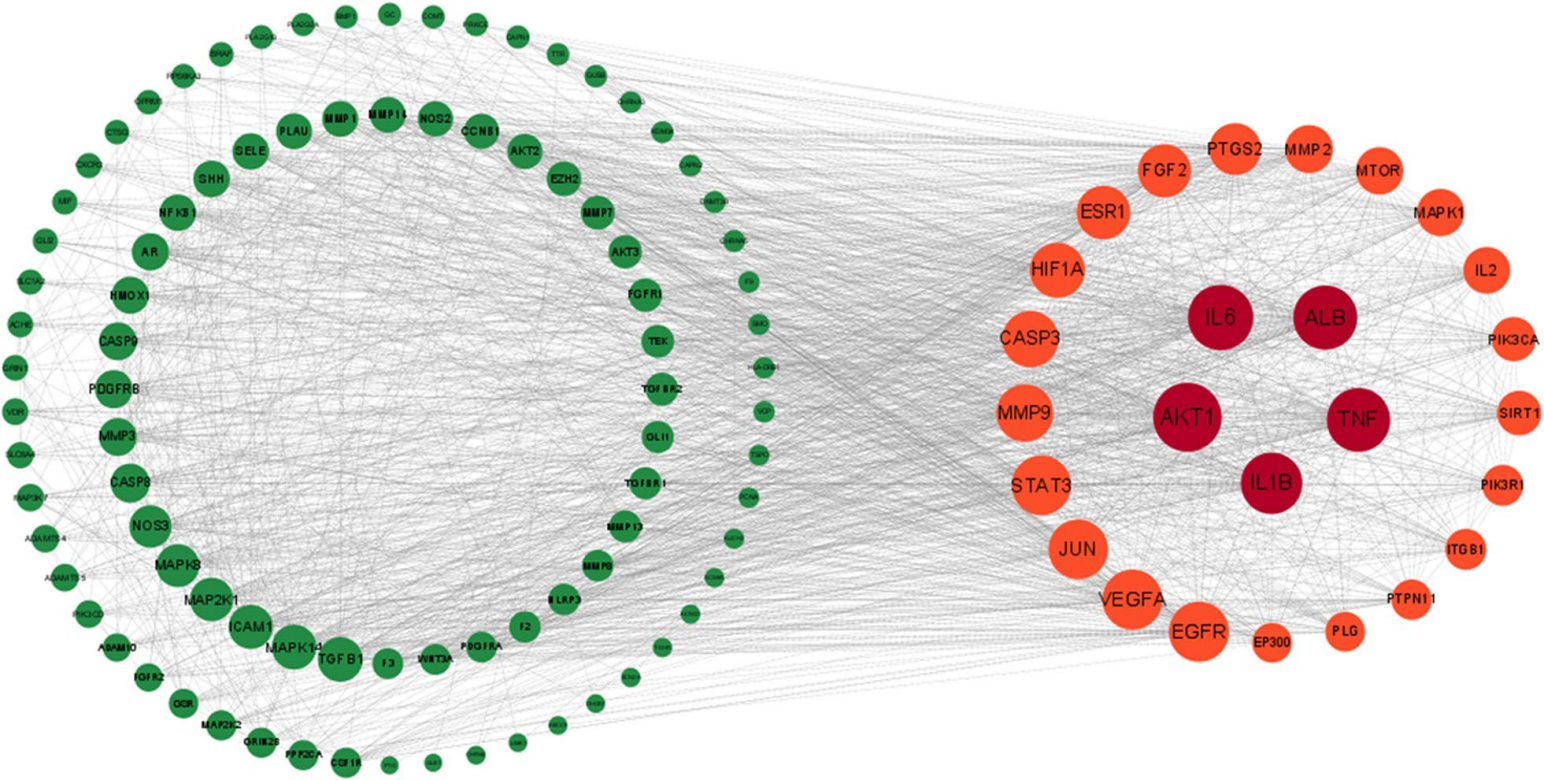
Drug–disease protein interaction (PPI) network diagram. The colour and size of the target point changes gradually according to the degree value. The higher the degree value, the larger the circle. As the degree value changes, the colour changes from green to red

The 118 potential targets were imported into the String database to build the model, and the relevant data were imported into Cytoscape 3.9.0 software to calculate the network topology parameters. By setting the parameters of closeness concentration (0.004–0.006), between concentration (116.552–1017.277) and degree (25.897–80.000), we finally obtained 26 core targets, of which AKT1, IL-6, ALB, TNF and IL-1β genes were the main targets (Fig. 2).

### Construction of drug-active ingredient-target network diagram

**Fig. 3:**
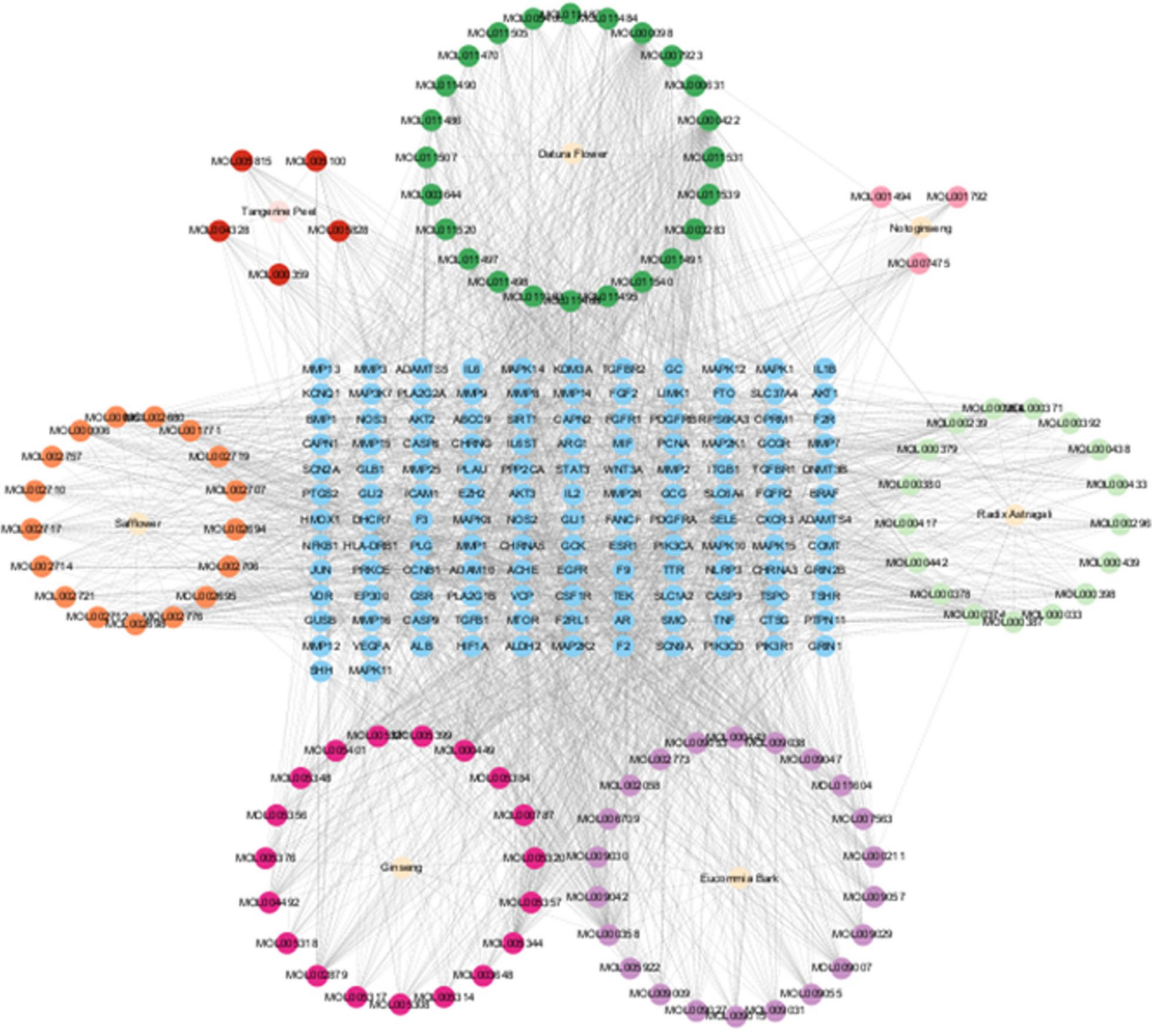
Drug-active ingredient-target network diagram. Reveal the relationship between drugs, active ingredients and targets

The composition data were imported into Cytoscape 3.9.0 software to produce the drug-active ingredient-target network diagram (Fig. 3). Topological analysis was performed to screen the main active components of the drug according to the degree value (Table 1).

**Table 1:** Top five main active ingredients

Molecule name	Mol ID	Degree	BC	CC
Kaempferol	MOL000422	85	384	0.001686
Quercetin	MOL000098	75	335	0.001675
Beta-sitosterol	MOL000358	64	402	0.001647
Stigmasterol	MOL000449	48	345	0.001647
Ginsenoside rh2	MOL005344	44	849	0.001692

### KEGG and GO enrichment analysis

**Fig. 4:**
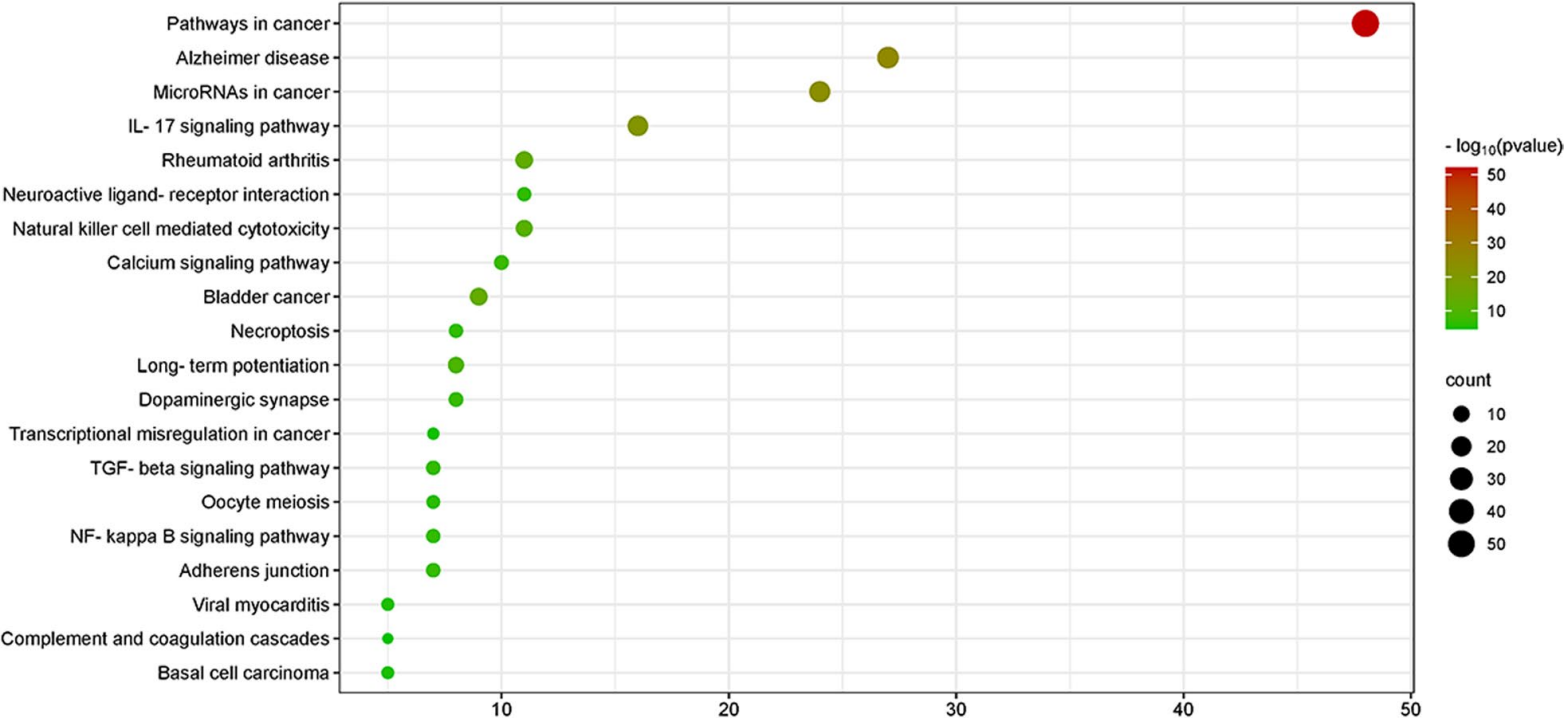
KEGG pathway enrichment analysis diagram. The size of the nodes represents the value of the degree. The horizontal axis in the figure represents the gene ratio

**Fig. 5:**
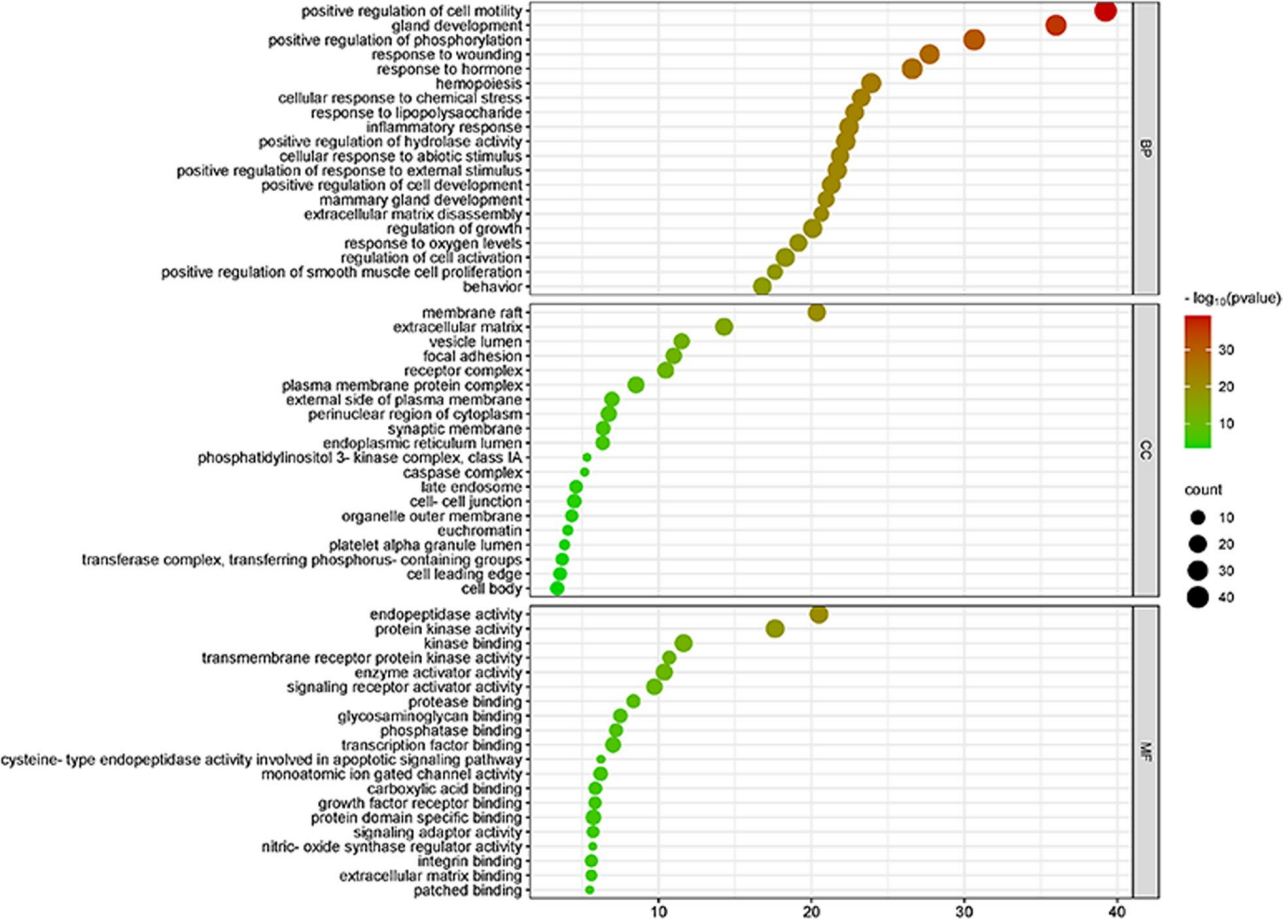
GO enrichment analysis diagram. The top 20 items of biological function are listed on the vertical axis, including GOMF, GOCC and GOBP, the horizontal axis in the figure represents the gene ratio. GO, Gene ontology, BP, biological process and CC, cell composition

The Metascape database was used for enrichment analysis, and the first 20 items of data were taken to make bubble charts. The KEGG enrichment analysis results showed that Osteoking mainly acts on LDH through pathways in cancer, Alzheimer disease, microRNAs in cancer, IL-17 signalling pathway and other pathways (Fig. 4). The results of GO biological process analysis showed that its biological processes were mainly involved in positive regulation of cell motility, gland development and positive regulation of phosphorylation. The cellular components mainly involved membrane raft, extracellular matrix and vesicle lumen. Molecular functions mainly include endopeptidase activity, protein kinase activity and kinase binding (Fig. 5).

### Molecular docking

**Fig. 6:**
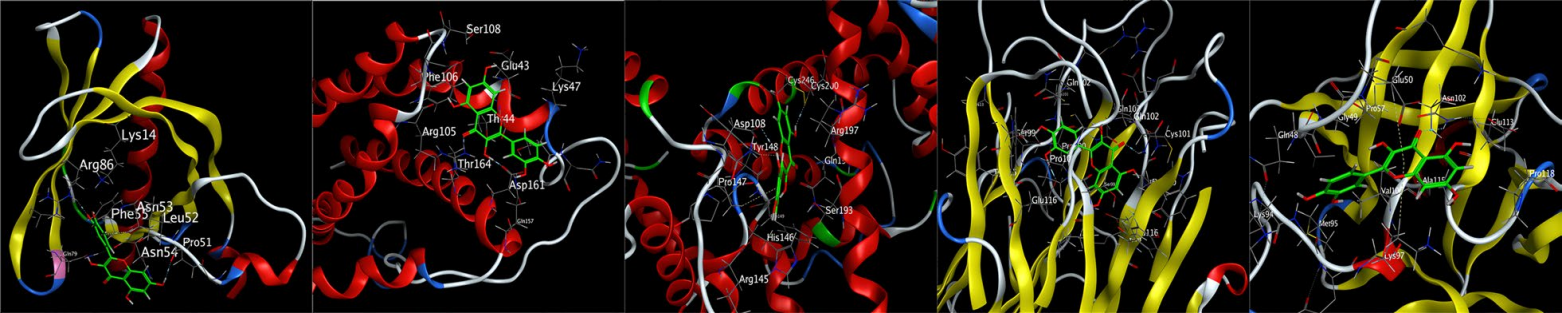
Molecular docking diagram. From left to right were kaempferol-AKT1, kaempferol-IL-6, kaempferol-ALB, kaempferol-TNF and kaempferol-IL-1β

**Fig. 7:**
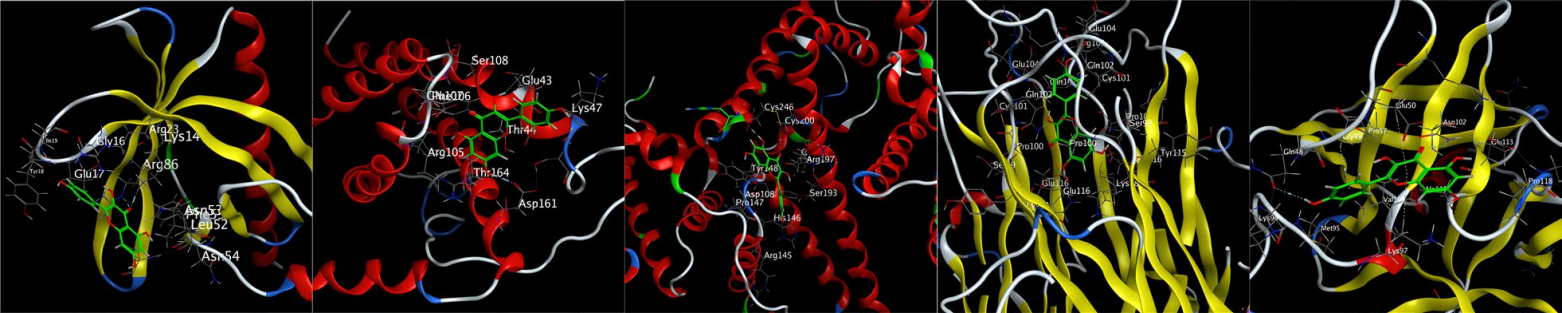
Molecular docking diagram. From left to right were quercetin-AKT1, quercetin-IL-6, quercetin-ALB, quercetin-TNF and quercetin-IL-1β

**Fig. 8:**
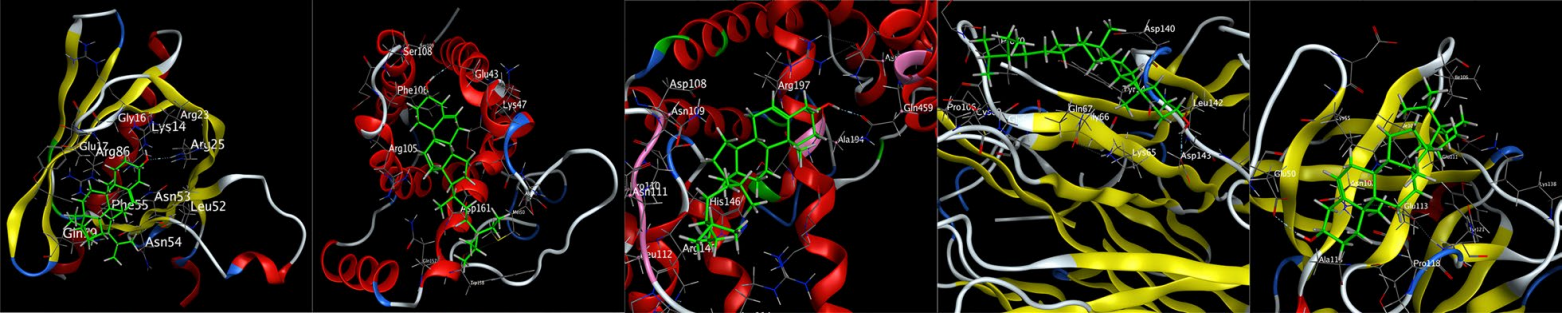
Molecular docking diagram. From left to right were beta-sitosterol-AKT1, beta-sitosterol-IL-6, beta-sitosterol-ALB, beta-sitosterol-TNF and beta-sitosterol-IL-1β

The first three main active components (kaempferol, quercetin and β-sitosterol) were molecular docked with the top five core targets (AKT1, IL-6, ALB, TNF and IL-1β). The results showed that the binding energies of the three active components to AKT1, IL-6, ALB, TNF and IL-1β were all < − 4.5 kcal/mol, and they could dock spontaneously. Among them, the binding energies of kaempferol to IL-6, ALB, TNF and IL-1β, quercetin to ALB, TNF and IL-1β and β-sitosterol to AKT1, IL-6, ALB, TNF and IL-1β were all < − 5 kcal/mol, indicating a good docking binding activity [15]. Among them, the docking activities of quercetin and ALB were the most significant (Table 2). Docking visualization is shown in Figs. 6, 7 and 8.

**Table 2:** Molecular docking information table

Targets	Molecule name	Mol ID	Binding energy (Kcal/mol)
AKT1	Kaempferol	MOL000422	− 4.73
	Quercetin	MOL000098	− 4.78
	Beta-sitosterol	MOL000358	− 5.27
IL-6	Kaempferol	MOL000422	− 5.29
	Quercetin	MOL000098	− 4.99
	Beta-sitosterol	MOL000358	− 6.00
ALB	Kaempferol	MOL000422	− 5.93
	Quercetin	MOL000098	− 6.06
	Beta-sitosterol	MOL000358	− 6.00
TNF	Kaempferol	MOL000422	− 5.55
	Quercetin	MOL000098	− 5.87
	Beta-sitosterol	MOL000358	− 5.57
IL-1β	Kaempferol	MOL000422	− 5.32
	Quercetin	MOL000098	− 5.31
	Beta-sitosterol	MOL000358	− 5.00

## Discussion

The results of the study showed that Osteoking had 26 core targets for LDH, mainly including AKT1, IL-6, ALB, TNF, IL-1β and other gene targets. Interleukin and tumour necrosis factor are considered to be the key mediators causing the aggravation of inflammatory process in low back pain due to LDH, with strong pro-inflammatory activity. IL-6, IL-1β and TNF-α are the most crucial ones, and they participate in a variety of inflammatory activities. IL-6, IL-1β and TNF-α show high levels in LDH, and their expression levels are affected by age and the degree of disc degeneration [16]. The signalling pathway of IL-1β can lead to acute-phase reactions, hypotension, vasodilation and fever. The results of IL-1 signal transduction can lead to up-regulation of adhesion molecules, lymphocyte recruitment and immune cell activation, further amplifying inflammation. Some scholars [17, 18] have found degenerative characteristics of the intervertebral disc, including annular rupture and degradation of the extracellular matrix of nucleus pulposus, after injection of TNF-α into the intervertebral disc of a pig model. These results suggest that TNF-α may be a key driver of disc degeneration. When TNF-α is stimulated, the level of substance *P* is increased, inducing the expressions of IL-1β, IL-6 and IL-8 [19], and aggravating the inflammatory response. As a key signalling node in the inflammatory response, AKT leads to the phosphorylation of its downstream signalling molecule mammalian target of rapamycin (mTOR), thereby promoting the polarization of giant cells and secreting IL-1, IL-6, TNF-α and other inflammatory factors to promote the inflammatory response [20, 21].

The KEGG and GO enrichment analysis results showed that the active components of Osteoking might exert such molecular functions as endopeptidase activity, protein kinase activity and kinase binding through such biological processes as positive regulation of cell motility, gland development, positive regulation of phosphorylation in membrane raft, extracellular matrix and vesicle lumen and then regulate pathways in cancer, Alzheimer disease, microRNAs in cancer, IL-17 signalling pathway and other pathways. MicroRNA is a single-stranded RNA with the size of about 21–23 bases. It regulates the secretion of cytokines and inflammatory factors by natural killer (NK) cells to participate in immune regulation and also mediates the remodelling of extracellular matrix, participating in many biological processes. In 2017, Hu [22] first linked microRNA with intervertebral discs and found that microRNA could attenuate the biosynthesis of chondroitin sulphate and regulate the degeneration of intervertebral discs. Subsequently, Kong [23] also proved that microRNA could act on human THP-4 cells and inhibit the inflammatory response in nucleus pulposus cells by regulating TNF receptor-related factor 6 (TRAF6). The infiltration and expression of IL-17 in the intervertebral disc tissue have a significant effect on local inflammation and radicular pain [24]. IL-17 expression in the intervertebral disc tissue is also at a high level, and together with interferon-γ (IFN-γ) and tumour necrosis factor (TNF-α), it is an important factor for regulating the inflammatory response in intervertebral disc protrusion [25]. Although cancer pathway and Alzheimer's disease pathway play an important role in the occurrence and development of LDH, the current research is still defective, and the mechanism of LDH is still unclear, which needs further investigation.

In this study, to verify the reliability of prediction targets and signalling pathways, three components with the highest activity and five core targets with the highest degree value were selected for molecular docking. The results showed that kaempferol, quercetin and β-sitosterol could dock well with AKT1, IL-6, ALB, TNF and IL-1β targets, and quercetin, β-sitosterol and ALB and β-sitosterol and IL-6 had strong docking activity (binding energy < − 6.0 kcal/mol), further verifying the reliability of the results.

In summary, Osteoking has multiple active components, among which kaempferol, quercetin and β-sitosterol have the most prominent activities, and can be used as multiple key targets for LDH, with good binding activity between the active components and the key targets. It can act on LDH through multiple targets and multiple pathways to relieve the symptoms.

## Data Availability

Publicly available datasets were analysed in this study. All data can be obtained in the related websites marked in this paper.
